# Individualised Positive End‐Expiratory Pressure During Robotic‐Assisted Radical Prostatectomy Guided by Intratidal Compliance–Volume Curve Analysis

**DOI:** 10.1111/aas.70067

**Published:** 2025-06-04

**Authors:** Christian M. Brandt, Silke Borgmann, Nino Frank, Johannes Spaeth, Johannes Hell, Sara Lozano‐Zahonero, Steffen Wirth, Stefan Schumann

**Affiliations:** ^1^ Department of Anesthesiology and Critical Care Medical Center – University of Freiburg, Faculty of Medicine, University of Freiburg Freiburg Germany

**Keywords:** electrical impedance tomography, individualised PEEP, intratidal compliance–volume curve analysis, positive end‐expiratory pressure ventilation, regional ventilation, robotic‐assisted radical prostatectomy

## Abstract

**Background:**

Steep Trendelenburg positioning and capnoperitoneum during robotic‐assisted prostatectomy adversely affect respiratory system mechanics and decrease dorsal lung ventilation.

**Objective:**

We hypothesised that individualising positive end‐expiratory pressure (PEEP) based on intratidal compliance–volume curve analysis enhances dorsal regional lung ventilation.

**Methods:**

Thirty male patients undergoing robotic‐assisted prostatectomy received a tidal volume of 7 mL kg^−1^ and a PEEP of 7 cmH_2_O. PEEP was increased in steps of 3 cmH_2_O until a predominance of horizontal intratidal compliance profiles occurred or a PEEP of 22 cmH_2_O was reached. The primary endpoint was the share of dorsal lung ventilation. Secondary endpoints included respiratory system mechanics, oxygenation, and haemodynamics.

**Results:**

Best PEEP was 20.6 ± 2.7 cmH_2_O. At best PEEP, defined as a predominance of horizontal intratidal compliance profiles, the share of dorsal ventilation was increased by 41% compared with PEEP 7 cmH_2_O (*p* < 0.001) and oxygenation improved [*P*_a_O_2_/F_i_O_2_ 417 mmHg (95% CI 387–446) vs. 400 mmHg (95% CI 365–436), *p* = 0.016]. Aside from a slight increase in norepinephrine dosage [0.054 μg kg^−1^ min^−1^ (95% CI 0.043–0.066) vs. 0.048 μg kg^−1^ (95% CI 0.037–0.059), *p* = 0.01], haemodynamic parameters were not affected by PEEP.

**Conclusion:**

Individualised PEEP guided by intratidal compliance–volume curve analysis enhanced dorsal lung ventilation, reduced driving pressure, and improved oxygenation during robotic‐assisted prostatectomy.

**Trial Registration:** DRKS00021009 (ClinicalTrials.gov identifier: DRKS00021009)

## 1 Introduction

Robotic‐assisted radical prostatectomy (RARP) has become the leading procedure for prostate cancer and is associated with reduced postoperative pain, shorter hospital stay and lesser need for blood transfusion than open surgery [1, 2]. At the same time, the required steep Trendelenburg position and capnoperitoneum impair respiratory system mechanics and expose patients to the risk of postoperative pulmonary complications [3, 4, 5, 6]. Whilst positive end‐expiratory pressure (PEEP) counteracts these adverse effects, the optimal PEEP and its method of determination during RARP remain unclear. Previous studies comparing high versus low PEEP during RARP showed inconsistent results [4, 7, 8, 9, 10]. More recent approaches on individualised PEEP decrease driving pressure, enhance end‐expiratory lung volume (EELV) and improve oxygenation by analysis of electrical impedance tomography (EIT), oesophageal pressure or driving pressure [3, 11, 12, 13]. However, for technical and personnel efforts, EIT and measurement of oesophageal pressure would not be applicable in every patient, and individualising PEEP based on driving pressure would require regular measurement manoeuvres.

We hypothesised that during RARP, a PEEP of 7 cmH_2_O is insufficient to prevent tidal recruitment/derecruitment and that individualising PEEP based on real‐time analysis of intratidal compliance–volume curves improves dorsal ventilation. Therefore, we investigated the respiratory system's recruitment/derecruitment state in patients undergoing RARP by applying intratidal compliance analysis for PEEP individualisation whilst using EIT to assess ventilation distribution.

## 2 Methods

This prospective clinical study was conducted at the University Medical Center Freiburg between April and July 2020. Approval from the local ethics committee (Ethik‐Komission der Albert‐Ludwigs‐Universität Freiburg, Chairperson Prof. Dr. R. Korinthenberg) was granted on 31 October 2019 (EK332/19), and the project was registered in the WHO‐listed German Trials Registry (trial registration number: DRKS00021009, principle investigator: Steffen Wirth, date of registration: 11 March 2020) before inclusion of the first patient. This study was conducted in accordance with the applicable guidelines and regulations, including the revised version of the Declaration of Helsinki of the World Medical Association, the applicable federal and state laws, and the medical professional regulations of the Federal Republic of Germany. Following preoperative anaesthesia evaluation, all patients were informed about the possibility of study participation. Written informed consent was obtained from all patients prior to participation. This manuscript adheres to the TREND guidelines.

### 2.1 Inclusion Criteria

Male patients aged > 18 years, undergoing elective RARP were eligible for inclusion.

### 2.2 Exclusion Criteria

Exclusion criteria were BMI ≥ 30 kg m^−2^, known lung disease requiring bronchodilator medication, American Society of Anesthesiologists (ASA) physical status > 3 or active cardiac implants.

### 2.3 Anaesthesia

Anaesthesia was induced using sufentanil (0.3–0.5 μg kg^−1^) and a target‐controlled infusion of propofol (Schnider model, 6–8 μg mL^−1^ effect‐site target concentration). Cisatracurium besylate (0.1–0.15 mg kg^−1^) was administered to achieve a sufficient level of relaxation, defined by a train‐of‐four ratio of 0/4 (NS 252 nerve stimulator, Fisher & Paykel). Subsequently, an 8.0 mm ID endotracheal tube was inserted into the trachea. Anaesthesia was maintained with a target bispectral index (BIS, Medtronic) of 40, using either sevoflurane, desflurane or TIVA at the discretion of the attending anaesthetist, who was not involved in the study team. Additionally, each patient received an infusion of remifentanil (0.1–0.2 μg kg^−1^ min^−1^). The radial artery was cannulised for continuous haemodynamic monitoring. Intravenous crystalloids and infusion of norepinephrine were administered at the discretion of the attending anaesthetist. Routine blood gas analyses, conducted by the attending anaesthetist, were synchronised with the study's schedule if possible.

### 2.4 Intraoperative Ventilation

Volume‐controlled ventilation with a tidal volume of 7 mL kg^−1^ predicted body weight [14] and PEEP 7 cmH_2_O was applied using a standard anaesthesia machine (Perseus A500, Draeger, Luebeck, Schleswig‐Holstein, Germany). The F_i_O_2_ was maintained at 0.6. The ventilation rate was adjusted to achieve an end‐expiratory CO_2_ partial pressure between 35 and 45 mmHg.

### 2.5 Study Protocol

**FIGURE 1:**
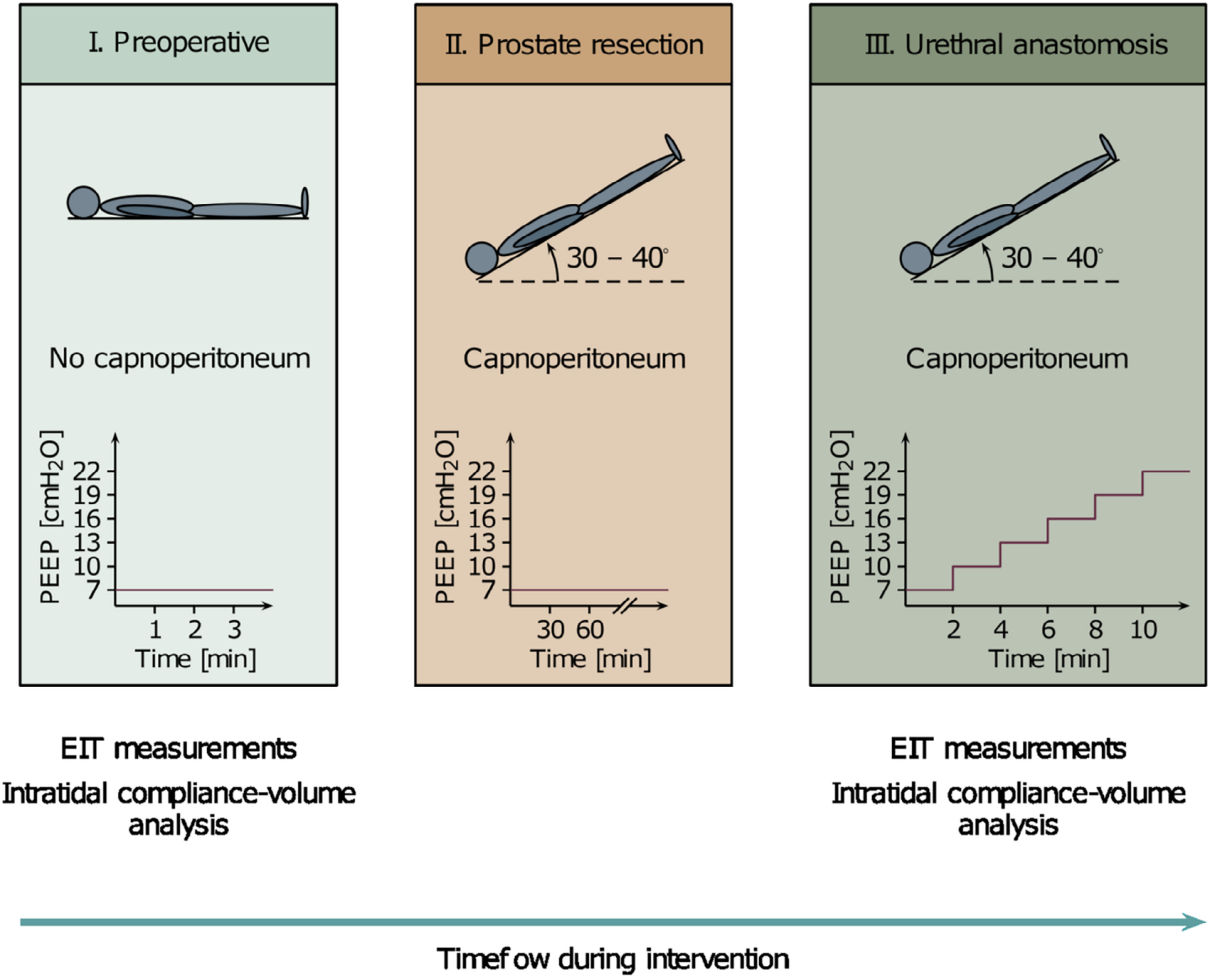
Illustration of the study protocol. After preparation of the experimental setup and the induction of anaesthesia, measurements were commenced in the horizontal position at a PEEP of 7 cmH_2_O (I). Subsequently, patients were rotated into the Trendelenburg position whilst maintaining a PEEP of 7 cmH_2_O during the prostate resection phase (II). The incremental PEEP trial was initiated with the beginning of the urethral anastomosis (III). The trial started with the baseline measurement at a PEEP of 7 cmH_2_O and extended until reaching the best PEEP according to intratidal compliance analysis or a maximum PEEP of 22 cmH_2_O. EIT, electrical impedance tomography.

An EIT electrode belt was placed around the thorax at the height of the fourth intercostal space. After establishing a 12 mmHg capnoperitoneum, the patient was placed in the Trendelenburg position, and surgery commenced. During urethral anastomosis, PEEP was increased from 7 cmH_2_O in steps of 3 cmH_2_O to individual best PEEP as defined by real‐time analysis of compliance–volume curves (see below) or a maximum of 22 cmH_2_O (Figure 1). Each PEEP step was thereby maintained for 2 min. Airway pressure and flow rates were recorded via the anaesthesia machine's serial communication interface (Medibus, Draeger). EIT images were captured at 20 frames per second using a PulmoVista 500 (Draeger). EIT measurements were performed whilst the patient was in a horizontal position at a PEEP of 7 cmH_2_O for at least 2 min and continuously in the Trendelenburg position during the PEEP trial. The intervention was delivered by the attending anaesthesiologist, whilst the on‐site evaluation of PEEP individualisation was conducted by the study personnel. Due to the study's design, no blinding was implemented.

### 2.6 Determination of Best PEEP Using Intratidal Compliance Analysis

On‐site, we used the Graphical User Interface for a model‐based decision support system for PEEP titration developed in our group [15]. This system determines the intratidal compliance–volume curves and categorises them according to the Gliding‐SLICE method [15, 16, 17]. In brief, the tidal volume of each breath was divided into 21 steps. Compliance for each volume step was calculated using multiple linear regression analysis of the data lying within the volume range surrounding the step by 1/6 of the tidal volume. The resulting intratidal compliance–volume curves were categorised into one of six compliance profiles (increasing [I], horizontal [H], decreasing [D] or a combination of these [IH, HD or IHD]). Whilst an increasing component of the profile indicates intratidal recruitment/derecruitment, a decreasing profile component indicates end‐tidal over‐distension. Consequently, the optimal respiratory mechanical conditions are represented by a horizontal compliance profile, indicating neither of the adverse conditions [17, 18, 19, 20, 21, 22, 23, 24]. The graphical user interface uses colour coding to visualise the predominant compliance profile, which can be easily interpreted by the user. Thus, the best individual PEEP was identified visually as the PEEP associated with a predominantly horizontal compliance profile or the maximum defined PEEP of 22 cmH_2_O according to the study specifications. In other words, even if there was still evidence of tidal recruitment, a maximum PEEP of 22 cmH_2_O was considered the best PEEP.

### 2.7 EIT

Functional tidal EIT images were calculated at baseline and each PEEP step [25]. In brief, impedance images corresponding to the beginning and the end of inspiration were identified and subtracted. The resulting functional tidal images were averaged over all breaths of a PEEP step, leading to a functional tidal image for each PEEP step and each patient. To exclude non‐lung regions, lung area was defined for each patient individually using the IDEAL method [26]. In brief, lung area was defined as the sum of pixels in the functional tidal images in horizontal position with tidal impedance of at least 20% of the respective maximum impedance.

The global inhomogeneity index was determined to quantify the inhomogeneity of ventilation distribution within the lung [27]. Ventral and dorsal regions were determined by bisecting the functional tidal images along the central horizontal axis, and the shares of ventral and dorsal ventilation were calculated [25]. The area of ventilated lung was determined as the number of ventilated pixels within a functional tidal image [26] and separately for the ventral and dorsal parts of the image. Global impedance curves were calculated [25] and changes in EELV were calculated between PEEP 7 in the Trendelenburg position and with capnoperitoneum and best PEEP [28]. Changes in EELV were rescaled to millilitres, based on the alignment of tidal impedance changes to measured tidal volume [28].

### 2.8 Endpoints of the Study

The primary endpoint was dorsal lung ventilation. Secondary endpoints included measures of respiratory system mechanics, haemodynamic parameters (heart rate, systolic, diastolic and mean arterial pressure and norepinephrine dosage) and gas exchange.

### 2.9 Statistical Evaluation

At the time of initiation, no valid data on the primary endpoint were available. Therefore, sample size calculation was based on the estimation of a standardised effect size. Assuming a moderate effect of 0.5 and a *t*‐distribution for dependent samples, 27 patients were required to achieve a power of 0.8 at an *α*‐level of 0.05. Thus, the target sample size was determined as *n* = 30. All results are presented either as mean ± SD or with the 95% CI in square brackets unless stated otherwise. The data were analysed using Microsoft Excel (Version 2016, Microsoft Corporation, Redmont, Washington, USA) and MATLAB (Version R2021a and R2016a, The MathWorks Inc., Natick, Massachusetts, USA). Two‐tailed paired Student's *t*‐tests were performed on data sets with normally distributed differences and homogeneous variances. For data sets violating either criterion, Wilcoxon signed‐rank tests for paired samples were used. Normality of the sample differences was investigated using quantile–quantile plots, and homogeneity of variances was determined using two‐sample *F*‐tests. For all hypothesis tests, *p <* 0.05 was considered to indicate statistical significance. All obtained *p*‐values were corrected for multiple comparisons using the method of Benjamini and Hochberg [29].

## 3 Results

The enrolment of patients started on 9 April 2020. Of the 68 patients screened for study participation, 17 were excluded due to refusal to participate (*n* = 5), pulmonary disease (*n* = 5), BMI > 30 kg m^−2^ (*n* = 6) or unavailability of the study team (*n* = 1). Therefore, 51 patients were enrolled in this study. In 13 patients, the EIT belt, although positioned as recommended between the fourth and sixth intercostal space, was too caudal in the Trendelenburg position. For these patients, artefacts from diaphragmal movement rendered the EIT data unusable, leading to their exclusion. Another eight patients were excluded from the analysis due to missing data (*n* = 6), subcutaneous emphysema resulting from a dislocated trocar during the surgery (*n* = 1) or corrupt data files (*n* = 1). Patient enrolment is shown in Figure S1 and patient characteristics are presented in Table 1. No important adverse events due to the study's intervention were recorded.

**TABLE 1:** Patient characteristics and choice of anaesthesia for the study population (*n* = 30).

Variable	Result
Age (years)	66 ± 7.1
Height (cm)	178 ± 7.3
Weight (kg)	80 ± 10.1
BMI (kg m^−2^)	25 ± 2.4
Smoking (yes/no)	5 (16.7%)/25 (83.3%)
ASA‐PS 1/2/3	8 (26.7%)/18 (60%)/4 (13.3%)
Maintenance of anaesthesia	
Sevoflurane/desflurane/propofol	13 (43.3%)/14 (46.7%)/3 (10%)
Trendelenburg position angle (°)	36 ± 1.9
Total duration of surgery (min)	160 ± 31
Duration of anaesthetic induction up to PEEP trial (min)	176 ± 31

*Note:* Entries are presented as mean ± standard deviation or number (%).

Abbreviations: ASA‐PS, American Society of Anesthesiologists Physical Status; BMI, body mass index.

### 3.1 Respiratory Mechanics and Best PEEP

Only increasing, increasing‐horizontal or horizontal compliance profiles were observed during the intervention. At a PEEP of 7 cmH_2_O, without capnoperitoneum and before Trendelenburg positioning, the intratidal compliance analysis of all patients showed horizontal compliance profiles. Following Trendelenburg positioning, no individual showed horizontal compliance profiles at PEEP 7 or 10 cmH_2_O. The best PEEP was determined in 6.7% of patients (*n* = 2) at 13 cmH_2_O, in 10% (*n* = 3) at 16 cmH_2_O, in 13.3% (*n* = 4) at 19 cmH_2_O and in 33.3% (*n* = 10) at 22 cmH_2_O. The remaining 36.7% of patients (*n* = 11) showed predominantly increasing (*n* = 7) or increasing‐horizontal (*n* = 4) compliance profiles at PEEP 22 cmH_2_O, which was therefore determined to be their best PEEP. The mean best PEEP was 20.6 ± 2.7 cmH_2_O.

Following Trendelenburg positioning, dynamic compliance decreased, and ventilation frequency, airway pressure, plateau pressure, driving pressure and *P*_a_CO_2_ increased (all *p* < 0.001, Table 2). The airway pressure and plateau pressure increased with increasing PEEP (both *p* < 0.001). Driving pressure was significantly lower, and compliance was significantly higher at the best PEEP compared with PEEP 7 cmH_2_O (both *p* < 0.001).

**TABLE 2:** Intraoperative ventilation and haemodynamic variables of the study population (*n* = 30).

	Horizontal position, PEEP 7 cmH_2_O (H7)	Trendelenburg position, PEEP 7 cmH_2_O (T7)	Trendelenburg position, best PEEP (TB)	*p*, H7 vs. T7	*p*, T7 vs. TB	*p*, H7 vs. TB
Ventilation						
Tidal volume (mL)	511 [494, 528]	513 [469, 529]	513 [497, 530]	0.453	0.684	0.684
Ventilatory frequency (breaths min^−1^)	12.4 [11.9, 12.9]	17.5 [16.6, 18.5]^†^	17.5 [16.5, 18.5]^†^	< 0.001	1	< 0.001
Peak pressure (cmH_2_O)	16.1 [15.6, 16.6]	26.7 [25.7, 27.8]^†^	36.4 [35.2, 37.7]^†^ ^,^ ^‡^	< 0.001	< 0.001	< 0.001
Plateau pressure (cmH_2_O)	13.6 [13.2, 14.1]	23.3 [22.3, 24.2]^†^	33.2 [32.0, 34.4]^†^ ^,^ ^‡^	< 0.001	< 0.001	< 0.001
Driving pressure (cmH_2_O)	6.6 [6.1, 7.0]	16.2 [15.2, 17.2]^†^	12.7 [12.1, 13.3]^†^ ^,^ ^‡^	< 0.001	< 0.001	< 0.001
*C*_rs_ (mL cmH_2_O^−1^)	86.4 [78.2, 94.5]	37.3 [34.4, 40.2]^†^	44.8 [41.8, 47.7]^†^ ^,^ ^‡^	< 0.001	< 0.001	< 0.001
*P*_et_CO_2_ (mmHg)	37.4 [36.6, 38.2]	40.4 [38.5, 42.3]^†^	41.4 [39.4, 43.3]^†^ ^,^ ^‡^	0.017	0.040	0.003
*P*_a_CO_2_ (mmHg)	42.6 [41.6, 43.6]	47.7 [46.1, 49.3]^†^	49.5 [47.2, 51.8]^†^	< 0.001	0.088	< 0.001
Haemodynamic variables						
Heart rate (beats min^−1^)	52.9 [48.3, 57.5]	60.9 [56.7, 65.0]^†^	60.3 [55.7, 65.0]^†^	0.009	0.218	0.016
Systolic blood pressure (mmHg)	124.1 [119.8, 128.4]	120.1 [115.7, 124.4]	116.3 [111.9, 120.7]	0.212	0.170	0.052
Diastolic blood pressure (mmHg)	66.2 [63.2, 69.2]	73.7 [71.4, 76.0]^†^	74.1 [71.6, 76.6]^†^	0.002	0.772	0.004
MAP (mmHg)	86.0 [82.8, 89.2]	88.6 [86.3, 91.0]	87.7 [84.8, 90.7]	0.316	0.649	0.611
Norepinephrine (μg kg^−1^ min^−1^)	0.044 [0.035, 0.053]	0.048 [0.037, 0.059]	0.054 [0.043, 0.066]^‡^	0.613	0.010	0.179
Oxygenation						
SpO_2_ (%)	98.8 [98.3, 99.3]	98.6 [98.2, 99.0]	98.7 [98.3, 99.1]	0.373	0.088	0.783
*P*_a_O_2_/F_i_O_2_ (mmHg)	445 [411, 479]	400 [365, 436]^†^	417 [387, 446]^†^ ^,^ ^‡^	0.002	0.016	0.037
Bispectral index	37.7 [35.7, 39.6]	41.1 [39.2, 43.0]^†^	41.5 [39.9, 43.0]^†^	0.026	0.750	0.006

*Note:* Entries are presented as mean [95% CI]. Significance was determined using two‐sided paired Student's *t*‐test.

Abbreviations: *C*_rs_, respiratory system compliance; F_i_O_2_, fraction of inspired oxygen; H7, horizontal position at PEEP 7 cmH_2_O; MAP, mean arterial blood pressure; *P*_a_CO_2_, arterial partial pressure of carbon dioxide; *P*_a_O_2_, arterial partial pressure of oxygen; *P*_et_CO_2_, end‐tidal partial pressure of carbon dioxide; SpO_2_, peripheral capillary oxygen saturation; T7, PEEP 7 cmH_2_O after insufflation of capnoperitoneum and Trendelenburg positioning; TB, best PEEP after insufflation of capnoperitoneum and Trendelenburg positioning.

^†^ *p* < 0.05, compared with PEEP 7 cmH_2_O at horizontal position (H7).

^‡^ *p* < 0.05, compared with PEEP 7 cmH_2_O after insufflation of capnoperitoneum and Trendelenburg positioning (T7).

### 3.2 Haemodynamic Variables, Oxygenation and Depth of Anaesthesia

After Trendelenburg positioning, there was a significant change in heart rate (*p* = 0.009), diastolic blood pressure (*p* = 0.002) and depth of anaesthesia (*p* = 0.026, Table 2). During the incremental PEEP trial, no significant changes in heart rate, systolic, diastolic or mean arterial blood pressure, or depth of sedation were observed, but a significant increase in norepinephrine dosage (*p* = 0.010) was noted when comparing PEEP 7 cmH_2_O with best PEEP.

*P*_a_O_2_/F_i_O_2_ decreased by 10.1% after Trendelenburg positioning at PEEP 7 cmH_2_O (*p* = 0.002), and was re‐increased by 4.3% at best PEEP (*p* = 0.016, Table 2).

### 3.3 Analyses of EIT Data

**FIGURE 2:**
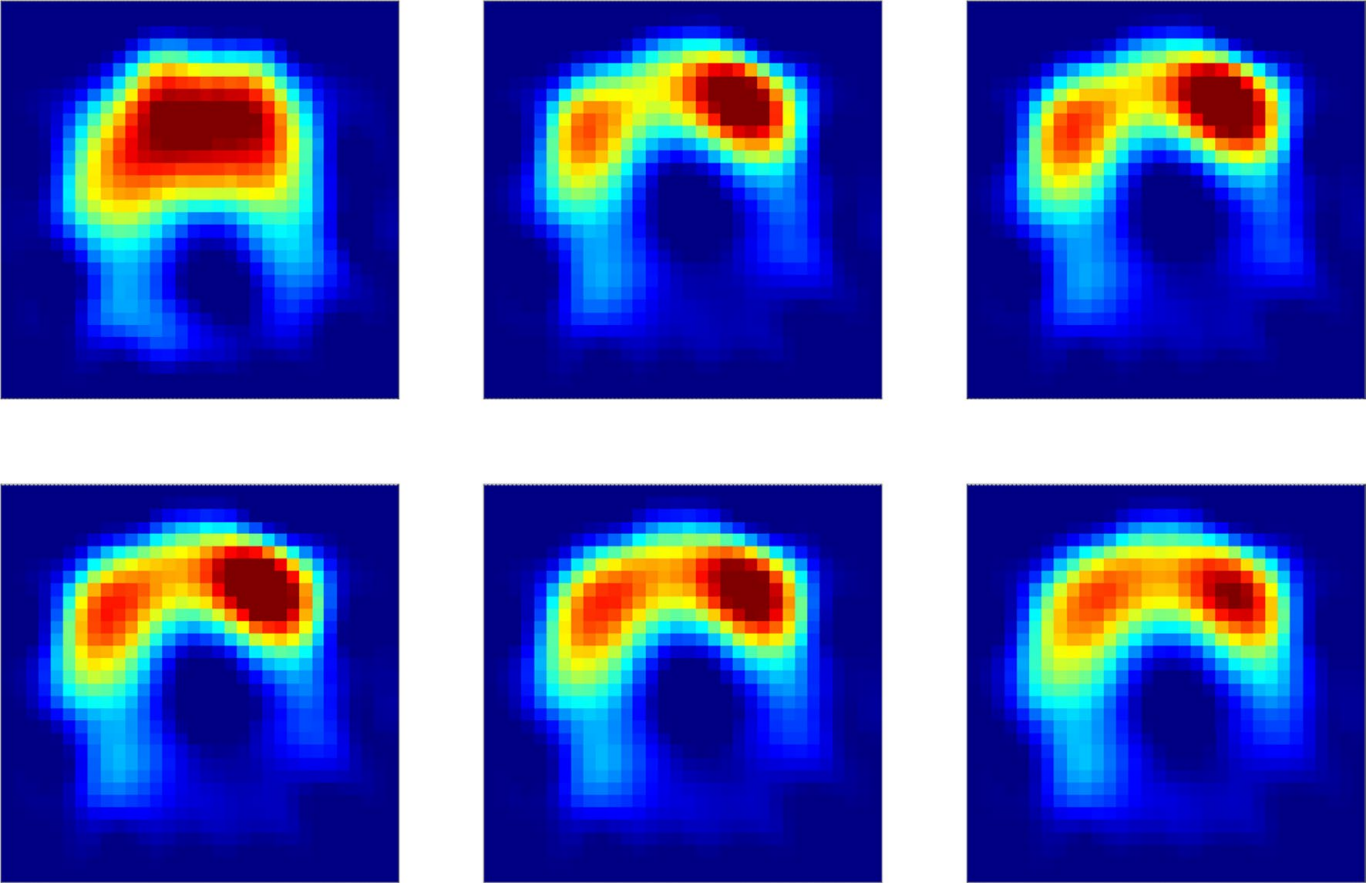
Exemplary functional EIT images from one patient for different PEEP steps and positions. The image on the upper left corresponds to a PEEP of 7 cmH_2_O in the horizontal position, and the image in the upper middle to a PEEP of 7 cmH_2_O in the Trendelenburg position. From the upper right through the lower row left to right, PEEP is increased in steps of 3 cmH_2_O whilst the patient remained in Trendelenburg positioning. The gain in ventilation in dorsal regions as well as the redistribution of air with higher PEEP steps is clearly visible.

**FIGURE 3:**
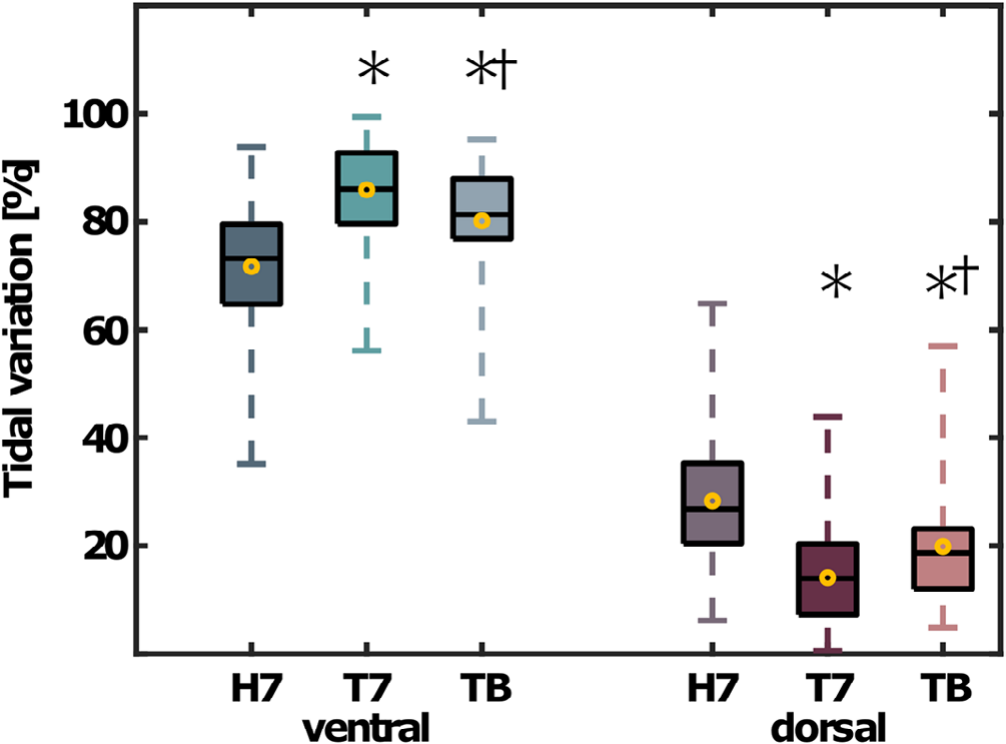
Tidal variation during different positioning and PEEP conditions (*n* = 30). Shares of ventral and dorsal ventilation during the horizontal position (H7), a PEEP of 7 cmH_2_O in the Trendelenburg position combined with capnoperitoneum (T7) and at the best PEEP (TB) under the same conditions. The boxes mark the interquartile range, the black lines the median and the yellow circles the mean values. The whiskers range from the zeroth to the fifth quartile. * marks a significant difference with respect to the horizontal position and † marks a significant difference with respect to the measurements at PEEP 7 cmH_2_O in the Trendelenburg position combined with capnoperitoneum. Significance was determined using two‐sided paired Student's *t*‐tests.

**FIGURE 4:**
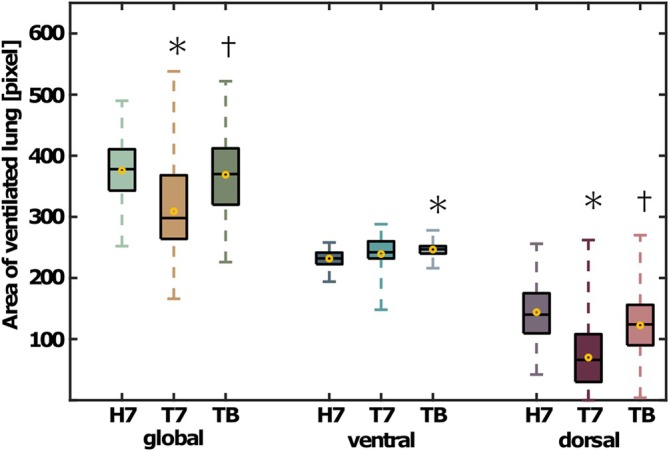
Area of ventilated lung (*n* = 30). Results for the horizontal position without capnoperitoneum (H7), the baseline at a PEEP of 7 cmH_2_O in the Trendelenburg position combined with capnoperitoneum (T7) and at the best PEEP under the same conditions (TB) are shown. The boxes mark the interquartile range, the black lines the median and the yellow circles the mean values. The whiskers range from the zeroth to the fifth quartile. * marks a significant difference with respect to the horizontal position and † marks a significant difference with respect to measurements at PEEP 7 cmH_2_O in the Trendelenburg position combined with capnoperitoneum. Significance was determined using two‐sided paired Student's *t*‐tests.

The share of dorsal ventilation was 28% [24, 33] in the horizontal position at a PEEP of 7 cmH_2_O. The share of dorsal ventilation decreased by 50% after Trendelenburg positioning at PEEP 7 cmH_2_O. At the best PEEP, the share of dorsal ventilation re‐improved by 41% (all *p* < 0.001). The ventral share of ventilation behaved inversely compared with the dorsal share of ventilation (Figures 2 and 3). The area of ventilated lung was smaller at PEEP 7 cmH_2_O in Trendelenburg (309 pixels [281, 337]) compared with horizontal position (372 pixels [352, 393], *p* < 0.001, Figure 4). At the best PEEP, the area of ventilated lung was 369 pixels [344, 394], which was comparable to the horizontal position at PEEP 7 cmH_2_O (*p* = 0.831).

The area of ventilated lung in the dorsal half of the image decreased after Trendelenburg positioning (70 pixels [50, 90]) at PEEP 7 cmH_2_O compared with the horizontal position (141 pixels [124,159], *p* < 0.001, Figure 4) and re‐increased at best PEEP to a level comparable to the horizontal position (122 pixels [100, 145], *p* = 0.125). The area of ventilated lung in the ventral half of the image after Trendelenburg positioning at PEEP 7 was comparable to that in the horizontal position (239 pixels [228, 251] vs. 231 pixels [226, 236], *p* = 0.072) and increased at the best PEEP compared with that in the horizontal position (247 pixels [242, 251], *p* < 0.001).

**FIGURE 5:**
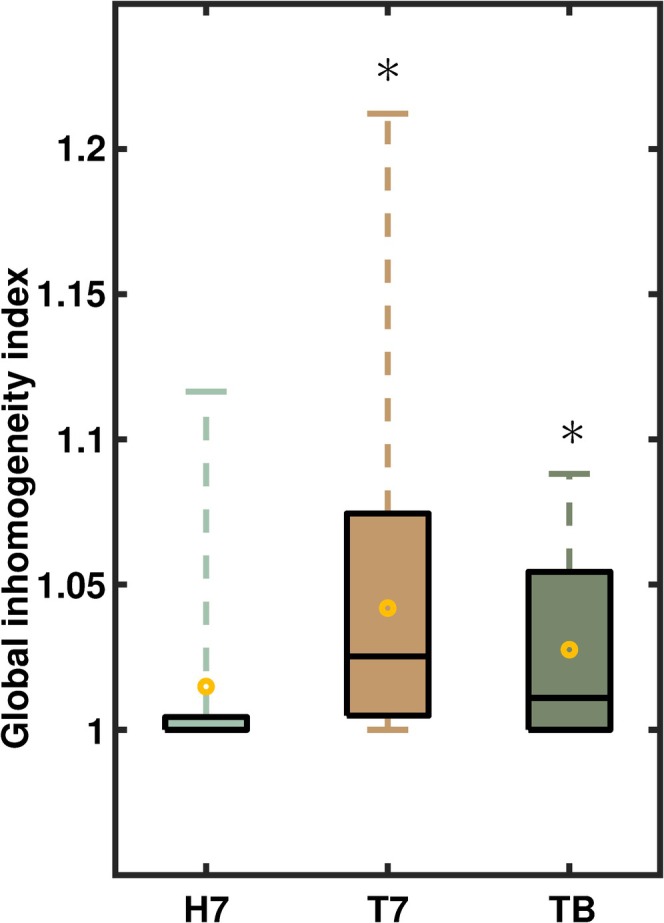
Global inhomogeneity index (*n* = 30). Results for the horizontal position without capnoperitoneum (H7), the baseline at a PEEP of 7 cmH_2_O in the Trendelenburg position combined with capnoperitoneum (T7) and at the best PEEP under the same conditions (TB) are shown. The boxes mark the interquartile range, the black lines the median and the yellow circles the mean values. The whiskers range from the zeroth to the fifth quartile. * marks a significant difference with respect to the horizontal position. Significance was determined using two‐sided paired Student's *t*‐tests.

The global inhomogeneity index increased from 1.01 [1.00, 1.03] to 1.04 [1.03, 1.06] after Trendelenburg positioning (*p* = 0.004, Figure 5). At best PEEP, the global inhomogeneity index was 1.03 [1.02, 1.04] and did not differ significantly from PEEP 7 cmH_2_O in the Trendelenburg position (*p* = 0.058) but was significantly different compared to PEEP 7 cmH_2_O in the horizontal position (*p* = 0.012). In the Trendelenburg position, the EELV increased by 804 ± 260 mL between PEEP 7 cmH_2_O and the best PEEP.

## 4 Discussion

During Trendelenburg position and capnoperitoneum, PEEP individualization using intratidal compliance–volume curve analysis improved dorsal ventilation, respiratory system compliance, driving pressure and oxygenation, whilst heart rate and blood pressure remained stable with only a slight increase in norepinephrine dosage.

### 4.1 Lung‐Protective Ventilation During RARP: Standardised Versus Individualised PEEP

The observed alterations in respiratory mechanics confirm previous findings of a pronounced decrease in dorsal ventilation following Trendelenburg positioning and capnoperitoneum [3, 4, 30]. The main cause is an accentuated dorsal cephalad diaphragm displacement due to elevated intra‐abdominal pressure, which puts patients at risk of postoperative pulmonary complications [4, 5, 6, 30, 31]. Consequently, lung‐protective ventilation appears crucial as it can decrease the incidence of postoperative pulmonary complications in lung‐healthy individuals [32]. Similarly, during RARP, lung‐protective ventilation results in fewer postoperative pulmonary complications than ventilation with high tidal volumes and very low or zero PEEP [8, 9, 10, 33]. However, disagreement persists on the optimal PEEP during RARP. Traditional approaches compare ‘low’ versus ‘high’ PEEP [4, 9, 10]. Different definitions of ‘low’ and ‘high’ are used, and the ‘optimal PEEP’ shows considerable variability. Exemplarily, Cheng et al. identified a PEEP of 5 cmH_2_O superior to 10 cmH_2_O based on oxygenation [10]. Contrarily, Shono et al. found a PEEP of 15 cmH_2_O to yield better oxygenation, more homogenous ventilation and a lower driving pressure compared to a PEEP of 5 cmH_2_O [4].

Whilst there is growing evidence for the beneficial effects of individualised PEEP titration during general anaesthesia [34, 35], recent studies indicate that particularly during RARP, this strategy may reduce postoperative pulmonary complications [12, 13].

### 4.2 Individualised PEEP Approaches and Recruitment Manoeuvres

One approach for PEEP individualisation during RARP utilises EIT, improving oxygenation and EELV [3]. However, this approach has drawbacks due to EIT's sensitivity to electrocautery and the need for initial recruitment manoeuvers [3, 36]. Whilst recruitment manoeuvers can improve oxygenation and compliance in the short term [37], there is no consistent evidence for a reduction in postoperative pulmonary complications [38] whilst at the same time, with peak airway pressures up to 40–50 cmH_2_O, both over‐distension and haemodynamic instability are risked [3, 36]. In ARDS patients, the 2023 ESICM guidelines suggest against recruitment manoeuvers due to increased mortality [39, 40].

Another approach is PEEP guidance by driving or oesophageal pressure, which reduced driving pressure and improved gas exchange compared to PEEP of 5 cmH_2_O in a recent study [11]. However, oesophageal pressure data require invasive monitoring and neither strategy indicates intratidal over‐distension, whilst again recruitment manoeuvres are required.

Therefore, real‐time breath‐wise compliance–volume analysis appears feasible for individual incremental PEEP titration during RARP, which led to the present study protocol excluding recruitment manoeuvres to minimise the risk of both pulmonary over‐distension and haemodynamic instability and avoid downsides of the other approaches at the same time.

### 4.3 Respiratory Mechanics

To our knowledge, only a few studies have individualised PEEP during RARP with a potential PEEP exceeding 15 cmH_2_O. Driving pressure‐guided PEEP titration led to a median ‘optimal’ PEEP of 18 cmH_2_O in the study of Boesing et al. [11]. Comparable to our results, they reported a plateau pressure of 29.4 ± 4.0 cmH_2_O. Girrbach et al. reported a lower plateau pressure of 26.4 ± 4.4 cmH_2_O in combination with a lower individualised PEEP of 14.7 ± 3.3 cmH_2_O guided by EIT [3]. However, individualisation of PEEP after Trendelenburg positioning but before insufflation of the capnoperitoneum and the 30° Trendelenburg positioning which was milder than in our study might account for this.

It is to note that during pneumoperitoneum, elevated plateau airway pressure can mainly be attributed to increased intra‐abdominal and pleural pressures and does not correspond to an equivalent rise in transpulmonary pressure [41, 42]. Therefore, under the conditions of RARP, a plateau pressure above the recommended 30 cmH_2_O [40] appears justifiable from a respiratory mechanics perspective. This is further supported by the fact that none of our patients exhibited compliance profiles indicating intratidal over‐distension.

Moreover, driving pressure is increasingly considered a primary indicator of lung‐protective ventilation [40, 43, 44]. High driving pressure is associated with postoperative pulmonary complications [44]. In our study, PEEP individualization lowered driving pressure, increased dorsal lung ventilation and enhanced EELV, similar to the findings of Girrbach et al. [3]. Contrary to our findings, they also noted a more homogeneous ventilation. This difference, again, might be attributed to the absence of capnoperitoneum during their PEEP individualization. Early PEEP adjustment seems advisable to mitigate atelectasis development [45]. In our study, PEEP adjustment was initiated rather late during the urethral anastomosis phase to generate flawless EIT data. This may also contribute to explaining why our best PEEP values are comparatively higher than those in other studies. Therefore, early PEEP individualization may offer further potential for improvement, as late PEEP titration and prolonged ventilation with insufficient PEEP are likely to have favoured atelectasis formation. On the other hand, this approach ensured that the anaesthesia duration was not extended for study purposes and was chosen because the focus of the study was on the methodology of PEEP titration rather than postoperative pulmonary complications as the primary outcome.

In summary, our findings indicate that the optimal PEEP during RARP is significantly higher than commonly used, yet varies notably, emphasising the necessity of individualised ventilation.

### 4.4 Haemodynamic Variables and Oxygenation

The haemodynamic stability of blood pressure and heart rate during our PEEP individualisation with only a slight increase in norepinephrine dosage suggests that during RARP, notably higher levels of PEEP may be tolerable than those commonly used. However, we did not assess cardiac output, and individualised PEEP was not maintained throughout the entire duration of surgery; therefore, the potential adverse effects of maintaining a higher, individualised PEEP throughout the entire procedure cannot be excluded.

Two studies reported a stable cardiac output applying individualised PEEP during RARP [11, 46]. Contrarily, Girrbach et al. documented pharmacological intervention due to bradycardia in 65% of patients during recruitment manoeuvres and/or PEEP titration [3]. In the present study, no patient required such intervention. This may support the use of an incremental PEEP trial instead of a classical recruitment manoeuvre to minimise both pulmonary over‐distension and haemodynamic instability at the same time. Shono et al. observed a lower cardiac output and higher vasopressor dosage comparing PEEP 15 cmH_2_O to 5 cmH_2_O [4]. Boesing et al. also reported a reduction in cardiac output during oesophageal pressure‐guided PEEP titration [11]. Haemodynamic impairment due to PEEP must therefore always be anticipated.

The observed slight improvement in oxygenation after PEEP individualization aligns with other studies [3, 4, 11] and demonstrates that gas exchange can be enhanced through intratidal compliance–volume analysis without the need for additional measures. This minor improvement may be explained by the measurement being performed immediately after PEEP titration. Whilst the clinical significance of this change may be questionable, it is consistent with the reduced intratidal recruitment/derecruitment and enhanced dorsal ventilation observed. Whether this effect would become more pronounced with the maintenance of individualised PEEP throughout the entire procedure or have a positive impact on gas exchange during the postoperative phase remains uncertain and was not addressed in this study, highlighting the need for further investigation.

### 4.5 Limitations

At the time of study conceptualisation in 2019, there was a lack of studies investigating PEEP individualisation during RARP, and the optimal PEEP range remained unknown. Considering the extreme physiological conditions during RARP, as well as the clinical experience of the authors, the maximum PEEP for the study was arbitrarily set at 22 cmH_2_O. This was done to ensure that potential over‐distension could be detected through intratidal compliance analysis, whilst selecting a sufficiently high yet ethically justifiable PEEP. Retrospectively, it became apparent that a significant proportion of patients still did not reach their best PEEP within this range, suggesting that in these cases, from a respiratory mechanics perspective, higher PEEP would have been even more favourable. This likely influenced the results, as both the benefits of higher PEEP on dorsal ventilation and oxygenation and the potential haemodynamic side‐effects may have been underestimated, ultimately affecting the overall impact of PEEP individualisation in this study. Nevertheless, the maximum PEEP was still considered the best possible choice within the scope of this study, as it demonstrated improvement compared to lower PEEP.

Only the short‐term effect of individualised PEEP was examined, which was not sustained throughout the entire duration of RARP. The influence on postoperative pulmonary complications and cardiac output was not assessed. PEEP was individualised rather late during surgery, which is likely to have favoured atelectasis formation and might have contributed to a comparatively high best PEEP. However, our study protocol allowed us to minimise interference between the EIT and simultaneous electrocautery and to prevent prolonged anaesthesia for study purposes.

All patients received neuromuscular blockade along with a continuous infusion of remifentanil, but the continuation of the neuromuscular blockade during surgery was not standardised, which may have influenced the results, as the potential for spontaneous respiratory efforts could not be entirely excluded.

On‐site, the frequencies of the compliance profiles could only be estimated subjectively. However, our retrospective data analyses confirmed that during the on‐site determined best PEEP, horizontal compliance profiles were indeed predominant in each case.

## 5 Conclusions

Individualised PEEP guided by intratidal compliance–volume curve analysis consistently resulted in higher levels than conventional PEEP, enhancing dorsal lung ventilation, reducing driving pressure and improving oxygenation during RARP. At the same time, the stability of heart rate and blood pressure, with only a slight increase in norepinephrine dosage, suggests that higher PEEP levels than those commonly used may be haemodynamically tolerable in this setting. The intratidal compliance–volume analysis is presented as an alternative for PEEP individualisation, offering a potentially more feasible, cost‐effective and less artefact‐prone or less invasive method in the clinical setting compared to EIT or oesophageal pressure measurement.

## Author Contributions

C.M.B. helped with patient recruitment, data collection and data analysis as well as drafting of the manuscript. S.B. helped with data collection and data analysis as well as drafting of the manuscript. N.F. helped with patient recruitment, data collection and data analysis. J.S. and J.H. helped with data analysis and drafting of the manuscript. S.L.‐Z. helped with data collection and data analysis. S.W. helped with the conceptualisation and implementation of the study design. S.S. helped with the conceptualisation and implementation of the study design, data analysis and drafting of the manuscript.

All authors participated in critical revision of the manuscript for important intellectual content, approved the submitted version and agreed to be accountable for all aspects of the work.

## Ethics Statement

Approval from the local ethics committee (Ethik‐Komission der Albert‐Ludwigs‐Universität Freiburg) was granted on 31 October 2019 (EK332/19).

## Conflicts of Interest

The authors declare no conflicts of interest.

## Supporting information


**Figure S1.** CONSORT flowchart of patient enrolment.

## Data Availability

The data that support the findings of this study are available from the corresponding author upon reasonable request.
