# Supplementary figures and images for: Individualised Positive End‐Expiratory Pressure During Robotic‐Assisted Radical Prostatectomy Guided by Intratidal Compliance–Volume Curve Analysis
Source: Acta Anaesthesiol Scand. 2025 Jun 4;69(6):e70067. doi: 10.1111/aas.70067 (PMC12136937; doi:10.1111/aas.70067)

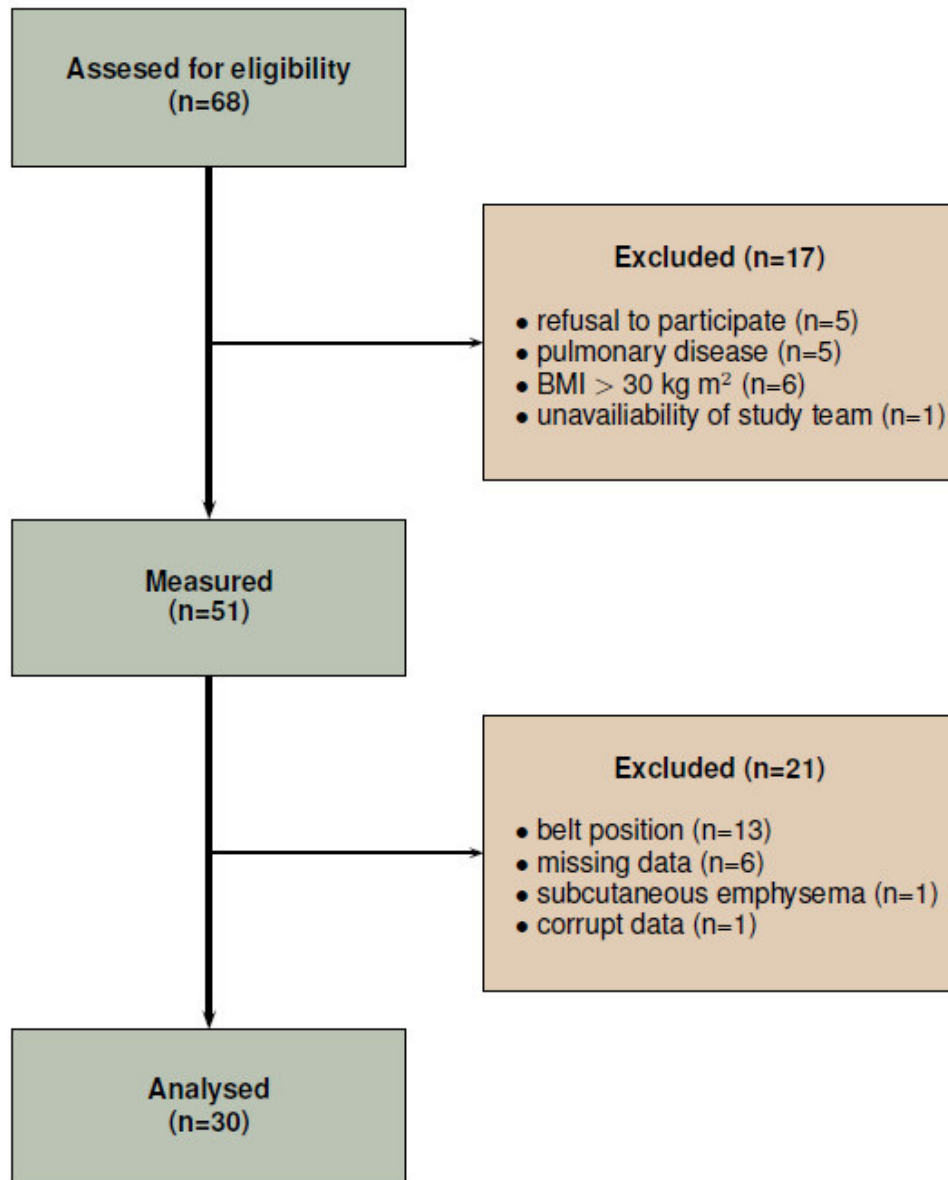

**Figure S1:** Flow chart of enrolment.

Supplement: Supplementary file 1 — Figure S1. CONSORT flowchart of patient enrolment. [file AAS-69-0-s001.pdf]
